# Risk of post-operative bleeding after dentoalveolar surgery in patients taking anticoagulants: a cohort study using the common data model

**DOI:** 10.1038/s41598-024-57881-7

**Published:** 2024-04-02

**Authors:** Joo-Yeon Lee, Seung-Hyun Park, Da-Mi Kim, Kyung-A. Ko, Jin-Young Park, Jung-Seok Lee, Ui-Won Jung, Jae-Kook Cha

**Affiliations:** Department of Periodontology, Research Institute for Periodontal Regeneration, Yonsei University College of Dentistry, 50 Yonsei-ro, Seodaemun-gu, Seoul, 03722 Republic of Korea

**Keywords:** Risk factors, Medical research, Dental patient assessment, Dentoalveolar surgery

## Abstract

This retrospective study aimed to determine risk factors associated with post-operative bleeding after dentoalveolar surgery in patients taking anticoagulants. Patients taking anticoagulants who were planned to undergo periodontal flap operation, tooth extraction or implant surgery were included. Patients were divided into two subgroups according to the maintenance of anticoagulants following medical consultation: (1) maintenance group and (2) discontinuation group. The analysed patient-related factors included systemic diseases, maintenance of anticoagulants and types of anticoagulant. Intra- and post-operative treatment-related factors, haemostatic methods and post-operative bleeding were collected for statistical analyses. There were 35 post-operative bleeding complications (6.5%) in the 537 included patients: 21 (8.6%) in maintenance group and 14 (4.8%) in discontinuation group. The type of anticoagulant (*p* = 0.037), tooth extraction combined with bone grafting (*p* = 0.016) and type of implant surgery (*p* = 0.032) were significantly related to the post-operative bleeding rate. In the maintenance group, atrial fibrillation [odds ratio (OR) = 6.051] and vitamin K inhibitors (OR = 3.679) were associated with a significantly higher bleeding risk. From this result, it can be inferred that the decision to continue anticoagulants should be made carefully based on the types of anticoagulant and the characteristics of dentoalveolar surgeries performed: extraction with bone grafting, multiple implantations and involvement of maxillary arch.

## Introduction

The number of patients undergoing dentoalveolar surgeries including periodontal flap operations, tooth extractions, and implant placements has increased recently, especially among individuals aged 65 to 74 years. This situation highlights the growing importance of considering various systemic diseases when performing dentoalveolar surgeries in the elderly population^1^.

As reported by the National Institute of Statistics and WHO, cardiovascular disease is the leading cause of death worldwide, with its prevalence highest among non-communicable diseases^2^. The long-term prescription of anticoagulants after cardiovascular surgeries is common for the treatment of conditions including atrial fibrillation, artificial heart valves, deep vein thrombosis, myocardial infarction and pulmonary embolism^3^. Therefore, various studies have investigated the risk of post-operative bleeding after dental surgeries where bleeding is expected in patients taking anticoagulants.

Three possible management methods for patients taking anticoagulants during dental surgical procedures have been reported: (1) maintaining the anticoagulants, (2) interrupting or reducing the anticoagulants, and (3) bridging with heparin^4^. The European Society of Cardiology guideline indicates that dental extractions of one to three teeth, periodontal surgeries and implant surgeries are low-risk interventions^5^. It has been reported that maintaining anticoagulant therapy during low-risk dental treatments does not increase the post-operative bleeding rate^5^.

In contrast to earlier reviews, several studies have shown that patients taking specific types of anticoagulants have a higher risk of bleeding compared with healthy individuals who are not taking medications^6,7^. A recent meta-analysis of post-operative bleeding in patients taking direct oral anticoagulants (DOACs) showed a statistically significant higher relative risk (RR = 3.04) compared to healthy individuals^8^. This suggests that continuing to take anticoagulants can increase the post-operative bleeding rate. Moreover, it is important to note that previous investigations on post-operative bleeding have primarily focused on teeth extraction, with few studies addressing other invasive treatments such as dental implant surgeries. Studies of implant surgeries tend to lead to similar conclusions that the post-operative bleeding rates are very low and fully controllable by local haemostatic measures regardless of the accompanying bone augmentation procedures, although these studies have only included small numbers of patients.

There has been little research into the correlation between the maintenance of anticoagulants and compliance with bleeding control across all dentoalveolar surgeries, including implant surgeries. Hence, to supplement the existing evidence, we hypothesized that among a large patient cohort taking anticoagulants in a single medical centre, the risk of bleeding complications would be significantly affected by several patient- and treatment-related factors, including the maintenance of anticoagulants. The current study aimed to identify risk factors associated with bleeding complications and to determine whether these factors lead to differences in bleeding complication rates.

## Methods

### Study design and population

This retrospective study was conducted using the Clinical Data Warehouse from the Severance Clinical Research Analysis Portal, Yonsei University Medical Center, Seoul, Republic of Korea (SCRAP 2.0). Overall patient data were standardized through the common data model provided by the SCRAP service and utilized in this study.

The following steps were used to determine the study population: first, all patients taking anticoagulants who visited Yonsei University Dental Hospital from January 2016 to June 2021 were selected. From this population, patients who were planned to undergo the following dental procedures where post-operative bleeding can occur were selected: periodontal flap operation, tooth extraction or implant surgery. Participants with non-drug-associated blood coagulation disorders such as haemophilia, factor deficiency, von Willebrand-Jürgens syndrome, liver cirrhosis or thrombocytopenia were excluded.

We then divided the patients into the following two subgroups according to the maintenance of anticoagulants as determined primarily based on their answers during medical consultations (*n* = 537):

1. Maintenance group (*n* = 245): participants who maintained anticoagulant therapy throughout the dental procedures.
2. Discontinuation group (*n* = 292): participants who discontinued anticoagulant therapy for 1 to 7 days according to the medical consultation request during the dental procedures.

The detailed dental histories recorded in electronic medical records were screened to collect potential risk factors that could cause bleeding complications after dental procedures. The types of anticoagulation agents were divided into the following three categories according to various clinical uses:

1. Platelet-aggregation inhibitors (PAIs).
2. Vitamin K inhibitors.
3. DOACs.

Demographic data such as age, sex and smoking history were also collected. Systemic internal conditions such as atrial fibrillation, artificial heart valves, deep vein thrombosis, myocardial infarction and pulmonary embolisms were recorded for each patient. The protocol of this study was approved by the Yonsei University Dental Hospital Institutional Review Committee (approval number 2-2021-0107).

### Surgical procedures

Surgical procedures were classified according to guidelines for the risk of bleeding after dental surgery in patients taking anticoagulants^9^. All of the participants underwent one of the surgical procedures performed by five experienced periodontists in the Department of Periodontology, and were advised to visit the emergency department if uncontrolled post-operative bleeding occurred:

1. Periodontal flap operation: periodontal flap surgery included intrasulcular incision, mucoperiosteal flap elevation, calculi removal using ultrasonic scalers, and granulation tissue removal using hand curettes. To examine the relationship between the extent of surgeries and the post-operative bleeding rate, a parameter related to the number of sextants involved (single or multiple sextants) was recorded.
2. Tooth extraction: tooth extractions were performed gently using extraction elevators and forceps, without flap elevation. The type of extraction varied depending on the number of teeth extracted (single or multiple extractions)^7^. Based on the decisions of the surgeons, a surgical approach including incision and minimal bone preparation was also utilized. Supplemental procedures such as bone grafting and the use of haemostatic fillers were recorded.
3. Implant surgery: single implantation referred to only a single implant being installed, while multiple implantations involved two or more implants being installed^6^. Data were also collected on whether ridge augmentation or sinus augmentation (using crestal or lateral approach) were accompanied.

### Anticoagulant management

All participants were receiving anticoagulation therapies, and they either continued or discontinued their anticoagulant medications primarily based on medical consultations with their attending physicians before receiving the dental treatments. The decision on whether to take the drug before and after surgery was made. For the patients who discontinued their anticoagulant medications, the drug was reinstated within 24 to 48 h after the dental treatment finished. Anticoagulant types including PAIs (98 types including aspirin), vitamin K inhibitors (warfarin and 3 other types) and DOACs (Xarelto and 4 other types) were extracted for data analyses.

### Local haemostatic measures

Local haemostatic measures were evaluated by a single experienced researcher (J.Y.L.) reading all of the dental history records and emergency-department records of the patients. The evaluated parameters included (1) intraoperative local haemostatic measures, (2) thromboembolic events, (3) post-operative bleeding events and (4) emergency-department records including haemostatic measures applied to patients with uncontrolled post-operative bleeding complications^7^.

Haemostatic measures were applied immediately after the dental procedures were completed in all patients. Patients with delayed bleeding in whom bleeding did not stop generally complained of bleeding during post-operative visits at 1 to 3 days after dental procedures, or they visited the emergency department due to bleeding complications. Additional haemostatic measures were applied to those who experienced post-operative bleeding events. One or more of the following measures were applied:

1. Intraoperative haemostatic method:
  1. Local wound compression using a bite swab for 30 minutes after all surgeries.
  2. During tooth extraction, absorbable atelocollagen sponge (Teruplug, Olympus Terumo Biomaterials Corporation, Tokyo, Japan) was inserted into the extraction socket before suturing.
  3. Suturing with resorbable Vicryl^®^ 4-0, 5-0 and 6-0 (Johnson & Johnson Medical, Norderstedt, Germany).
2. Post-operative haemostatic method for rebleeding participants:
  1. Local wound compression using a bite swab for 30 min.
  2. Suturing with resorbable Vicryl^®^ 4-0, 5-0 and 6-0 (Johnson & Johnson Medical).
  3. Haemostasis with infiltration anaesthesia (Huons^®^, lidocaine HCL 2% with epinephrine at 1:80,000).

For patients showing uneventful healing, the sutures were removed at 1–10 days after surgery according to the study protocol.

### Bleeding-related factors

The patient-related risk factors selected for comparison included systemic diseases, maintenance of anticoagulants, and anticoagulant types, while the treatment-related risk factors included surgical sites, types of surgery, supplemental procedures (i.e. bone grafting, use of haemostatic filler, ridge augmentation and sinus augmentation), and applied haemostatic measures.

### Statistical analyses

Statistical analyses were performed using R software (version 4.3.1, R Foundation for Statistical Computing, Vienna, Austria) to find out whether the bleeding-related risk factors were significantly associated with the post-operative bleeding frequency, which was the primary outcome of the present study. Normality of the data distribution was confirmed for a single patient-related factor (age) using the Shapiro–Wilk test (*p* > 0.05), while the other treatment-related factor (the number of teeth extracted) was found to be non-parametric. The other variables were included as categorical variables.

Intergroup comparisons of the categorical variables were performed using either the chi-square test or Fisher’s test. Continuous variables were quantified as mean ± SD values, and either the *t*-test or Mann–Whitney test was used for intergroup comparisons. The association of each variable with the post-operative bleeding rate was analysed using a univariate logistic regression model. All variables that were significantly associated with post-operative bleeding were included in a multiple logistic regression analysis.

Odds ratios (ORs) with 95% confidence intervals were calculated. The criterion for significance was set as *p* < 0.05, and post-hoc analyses were conducted for the categorical variables consisting of three or more levels.

### Ethics declarations and consent to participate

This study was approved by the Yonsei University Dental Hospital Institutional Review Committee (approval number 2-2021-0107). Informed consent of the participants was obtained in accordance with the relevant regulations of the Yonsei University Dental Hospital Institutional Review Board, with the anonymization of the patient data. All methods were performed following the relevant guidelines of the Institutional Review Board.

## Results

### Demographic data

**Figure 1:**
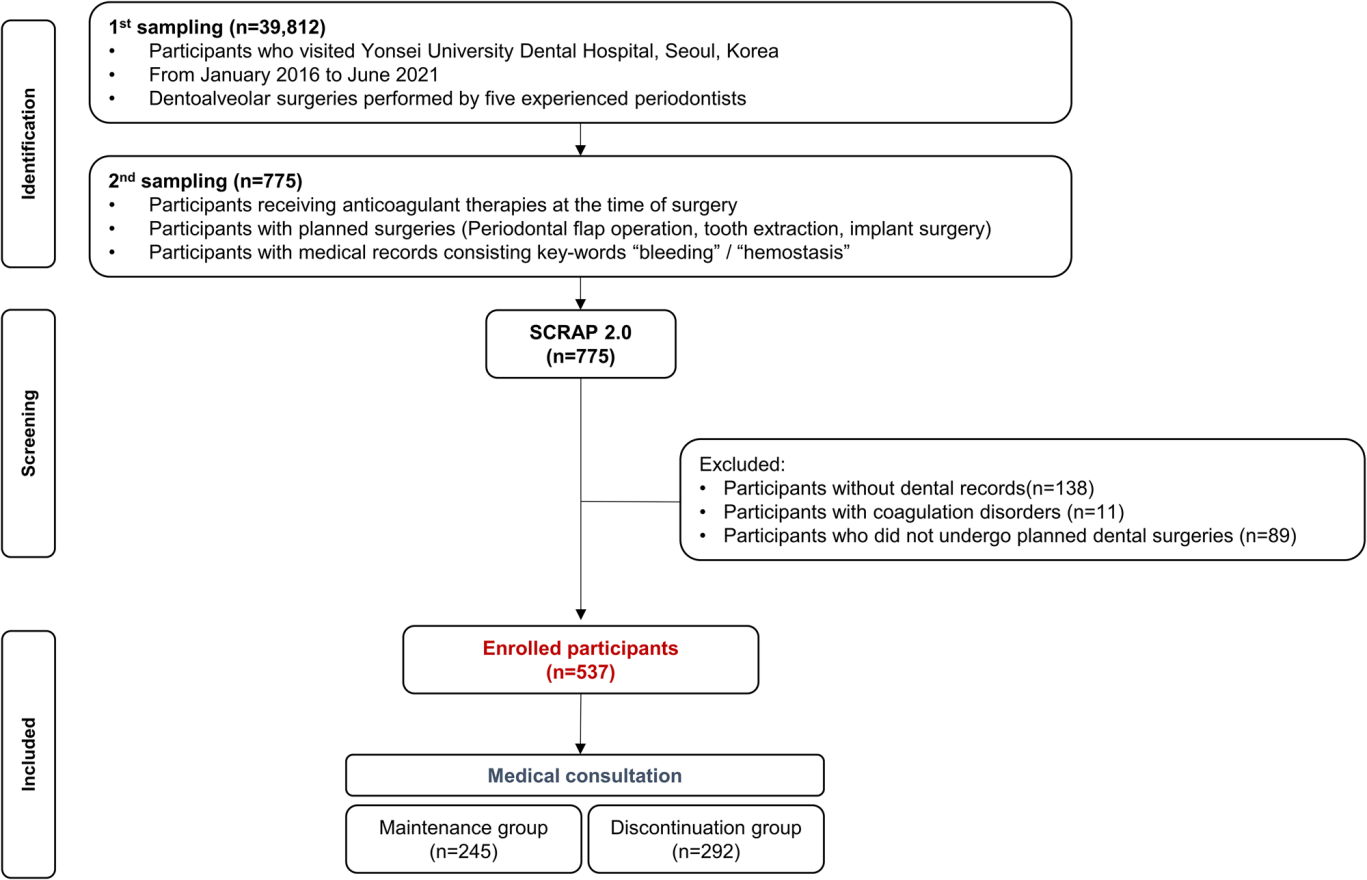
Flowchart of the study.

The concise baseline demographic data are presented in Table 1. The 537 included participants consisted of 300 (55.9%) males and 237 (44.1%) females aged 18–94 years (mean age: 71.0 ± 9.63 years). Anticoagulation therapy was maintained during dental surgery in 245 patients, and discontinued in the other 292 patients (Fig. 1). There were no significant differences between these two groups in age or sex (*p* > 0.05). In terms of systemic conditions, there were intergroup differences in the hypertension rate (*p* = 0.039) and the presence of artificial heart valves (*p* = 0.014).

**Table 1:** Summary of demographic characteristics of patients taking anticoagulants.

	Maintenance group (*n* = 245)	Discontinuation group (*n* = 292)	Total	*p*-value
Age (mean ± SD)	70.70 ± 9.28	71.26 ± 9.89	71.0 ± 9.63	0.504
Sex, *n* (%)				0.647
Male	140 (57.14)	160 (54.79)	300 (55.87)	
Female	105 (42.86)	132 (45.21)	237 (44.13)	
Systemic diseases, *n* (%)				
Hypertension	163 (66.53)	219 (75)	382 (71.14)	0.039*
Diabetes mellitus	76 (31.02)	101 (34.59)	177 (32.96)	0.433
Cerebrovascular diseases	27 (11.02)	37 (12.67)	64 (11.92)	0.650
Osteoporosis	37 (15.10)	43 (14.73)	80 (14.90)	0.999
Atrial fibrillation	16 (6.53)	29 (9.93)	45 (8.38)	0.208
Artificial heart valves	70 (28.57)	56 (19.18)	126 (23.46)	0.014*
Deep vein thrombosis	19 (7.76)	20 (6.85)	39 (7.26)	0.814
Myocardial infarction	10 (4.08)	24 (8.22)	34 (6.33)	0.075
Pulmonary embolisms	3 (1.22)	8 (2.74)	11 (20.05)	0.353
Anticoagular agents, *n* (%)				0.929
PAIs	222 (90.61)	267 (91.44)	489 (91.06)	
Vitamin K inhibitors	18 (7.35)	19 (6.51)	37 (6.89)	
DOACs	5 (2.04)	6 (2.05)	11 (2.05)	
Dental arch, *n* (%)				0.658
Mandibular arch	98 (40)	126 (43.15)	224 (41.71)	
Maxillary arch	132 (53.88)	152 (52.05)	284 (52.89)	
Both	15 (6.12)	14 (4.79)	29 (5.40)	
Type of teeth, *n* (%)				0.290
Anterior	36 (14.69)	54 (18.49)	90 (16.76)	
Posterior	209 (85.31)	238 (81.51)	447 (83.24)	
Type of surgery, *n* (%)				0.090
Flap operation	55 (22.45)	46 (15.75)	101 (18.81)	
Extraction	78 (31.84)	90 (30.82)	168 (31.28)	
Implant surgery	112 (45.71)	156 (53.42)	268 (49.91)	

*Statistically significant in chi-square test or fisher test (*p* < 0.05).

Overall demographic charateristics including treatment-related factors are demonstrated in Supplementary Table S1. It was shown that the treatment-related parameters did not differ significantly between the two subgroups.

### Post-operative bleeding events

There were 35 cases of post-operative bleeding events: 21 (60%) in the maintenance group and 14 (40%) in the discontinuation group (Table 2). No serious intraoperative complications occurred in any of the patients. However, four patients (two from each subgroup) were admitted to the emergency department due to bleeding complications (at a mean of 1.25 days after the planned surgeries). Additional bite swabs and infiltrative anaesthesia were applied as haemostatic procedures, and they were discharged after confirming haemostasis. Post-operative bleeding was controllable in all participants, with no fatal outcomes.

**Table 2:** Frequencies of post-operative bleeding complications according to patient and treatment-related factors.

	No complications (n = 502)	Complications (n = 35)	*p*-value
Age (mean ± SD)	71.15 ± 9.60	68.91 ± 9.57	0.195
Sex, *n* (%)			0.282
Male	284 (56.57)	16 (45.71)	
Female	218 (43.42)	19 (54.29)	
Smoking history, *n* (%)			0.154
None	474 (94.42)	31 (88.57)	
Former smoker	14 (2.79)	3 (8.57)	
Current smoker	14 (2.79)	1 (2.86)	
Systemic diseases, *n* (%)			
Hypertension	362 (72.11)	20 (57.14)	0.090
Diabetes mellitus	171 (34.06)	6 (17.14)	0.061
Cerebrovascular diseases	63 (12.54)	1 (2.86)	0.150
Osteoporosis	72 (14.34)	8 (22.86)	0.262
Atrial fibrillation	39 (7.77)	6 (17.14)	0.062
Artificial heart valves	116 (23.11)	10 (28.57)	0.595
Deep vein thrombosis	36 (7.17)	3 (8.57)	0.733
Myocardial infarction	31 (6.18)	3 (8.57)	0.479
Pulmonary embolisms	10 (1.99)	1 (2.86)	0.527
Anticoagular agents, *n* (%)			0.037*
PAIs	461 (91.83)	28 (80)	
Vitamin K inhibitors	32 (6.37)	5 (14.29)	
DOACs	9 (1.79)	2 (5.71)	
Continuation of medication, *n* (%)			0.112
Maintenance group	224 (44.62)	21 (60)	
Discontinuation group	278 (55.38)	14 (40)	
Dental arch, *n* (%)			0.132
Mandibular arch	215 (42.83)	9 (25.71)	
Maxillary arch	260 (51.79)	24 (68.57)	
Both	27 (5.38)	2 (5.71)	
Type of teeth, *n* (%)			0.864
Anterior	85 (16.93)	5 (14.29)	
Posterior	417 (83.07)	30 (85.71)	
Type of surgery, *n* (%)			0.807
Flap operation	93 (18.52)	8 (22.86)	
Extraction	158 (31.47)	10 (28.57)	
Implant surgery	251 (50)	17 (48.57)	
Periodontal flap operation (*n* = 101)			0.717
One sextant	69 (13.75)	6 (17.14)	
Multiple sextants	24 (4.78)	2 (5.71)	
Teeth extraction (*n* = 168)			
Type of teeth extraction			0.962
One tooth	101 (20.12)	6 (17.14)	
Multiple teeth	57 (11.35)	4 (11.43)	
Number of teeth involved (extraction) (mean ± SD)	1.81 ± 1.60	1.70 ± 1.00	0.756
Extraction with bone graft, *n* (%)			0.016*
Yes	12 (2.39)	4 (11.43)	
No	146 (29.08)	6 (17.14)	
Use of hemostatic filler, *n* (%)			0.457
Yes	76 (15.14)	3 (8.57)	
No	82 (16.33)	7 (20)	
Implant surgery (*n* = 268)			
Type of implant surgery, *n* (%)			0.032*
Single implantation	142 (28.29)	4 (11.43)	
Multiple implantation	109 (21.71)	13 (37.14)	
Combined sinus augmentation, *n* (%)			0.078
None	202 (40.24)	11 (31.43)	
Crestal approach	40 (7.97)	4 (11.43)	
Lateral approach	7 (1.39)	2 (5.71)	
Combined ridge augmentation, *n* (%)			0.238
Yes	159 (31.67)	15 (42.86)	
No	92 (18.33)	2 (5.71)	
Hemostatic measures, *n* (%)			0.603
Bite swab	38 (7.57)	3 (8.57)	
Hemostatic filler and suturing	76 (15.14)	3 (8.57)	
Bite swab and suturing	388 (77.29)	29 (82.86)	

*Statistically significant in chi-square test or fisher test (*p* < 0.05).

A statistically significant difference was observed in anticoagulant types, with PAIs showing the lowest bleeding rate (*p* = 0.037). Post-hoc analyses of anticoagulant types indicated that there were no statistically significant differences.

Regarding the treatment-related factors, periodontal flap operations were not significantly associated with post-operative bleeding, but significant effects were observed for extractions involving bone grafting (*p* = 0.016) and the type of implant surgery (*p* = 0.032). Single and multiple implantations showed significant differences in post-hoc analyses of the implant surgery type (*p* = 0.016), with multiple implantation showing a higher post-operative bleeding frequency.

### Univariate analyses of bleeding risk factors

The univariate analyses showed that diabetes mellitus (OR = 0.400,* p* = 0.046), involvement of the maxillary arch (OR = 2.205, *p* = 0.049) and extraction with bone grafting (OR = 5.269, *p* = 0.006) were significantly correlated with post-operative bleeding. The use of haemostatic filler did not show a statistically significant impact on the post-operative bleeding frequencies. Regarding implant surgery procedures, multiple implantations (OR = 1.663), combined sinus augmentation (OR = 4.483 via lateral approach; OR = 1.569 via crestal approach) and combined ridge augmentation (OR = 1.618) were also not significant (Table 3).

**Table 3:** Univariate analysis of post-operative bleeding events by potential risk factors.

	OR	95% Confidence Interval		*p*-value
		Lower	Upper	
Age	0.977	0.945	1.011	0.185
Sex				
Male	1 (Ref)			
Female	1.547	0.777	3.078	0.214
Smoking history				
None	1 (Ref)			
Former smoker	3.276	0.894	12.008	0.073
Current smoker	1.092	0.139	8.578	0.933
Presence of systemic diseases				
Hypertension	0.516	0.257	1.036	0.063
Diabetes mellitus	0.400	0.163	0.983	0.046*
Cerebrovascular diseases	0.205	0.028	1.523	0.121
Osteoporosis	1.770	0.774	4.048	0.176
Atrial fibrillation	2.456	0.962	6.274	0.060
Artificial heart valves	1.331	0.721	2.852	0.462
Deep vein thrombosis	1.214	0.354	4.156	0.758
Myocardial infarction	1.424	0.413	4.912	0.575
Pulmonary embolisms	1.447	0.180	11.639	0.728
Anticoagular agents				
PAIs	1 (Ref)			
Vitamin K inhibitors	2.573	0.931	7.111	0.069
DOACs	3.659	0.754	17.744	0.107
Continuation of medication	1.862	0.926	3.744	0.081
Dental arch				
Mandibular arch	1 (Ref)			
Maxillary arch	2.205	1.004	4.845	0.049*
Both	1.770	0.363	8.622	0.480
Type of teeth				
Anterior	1 (Ref)			
Posterior	1.223	0.461	3.243	0.686
Type of surgery				
Flap operation	1 (Ref)			
Extraction	0.736	0.280	1.930	0.533
Implant surgery	0.787	0.329	1.886	0.592
Periodontal flap operation				
Type of periodontal flap operation				
One sextant	1.317	0.525	3.307	0.557
Multiple sextants	1.262	0.283	5.624	0.760
Teeth extraction				
Type of teeth extraction				
One tooth	0.817	0.326	2.047	0.667
Multiple teeth	0.966	0.324	2.878	0.950
Number of teeth involved (extraction)	0.937	0.680	1.292	0.693
Extraction with bone graft	5.269	1.605	17.291	0.006*
Hemostatic filler	0.525	0.157	1.759	0.296
Implant surgery				
Type of implant surgery				
Single implantation	0.393	0.130	1.183	0.097
Multiple implantation	1.663	0.787	3.513	0.183
Combined sinus augmentation				
Crestal approach	1.569	0.525	4.686	0.420
Lateral approach	4.483	0.891	22.554	0.069
Combined ridge augmentation	1.618	0.807	3.243	0.175
Hemostatic measures				
Bite swab	1 (Ref)			
Hemostatic filler and suturing	0.500	0.096	2.595	0.409
Bite swab and suturing	0.947	0.275	3.254	0.931

For systemic conditions, a reference value of 1 was set for cases having no systemic diseases. Regarding the treatment-related parameters of each dentoalveolar surgery, cases where the surgery or additional procedure was not performed served as reference.

*Statistically significant (*p* < 0.05).

### Subgroup analyses of bleeding risk factors

**Figure 2:**
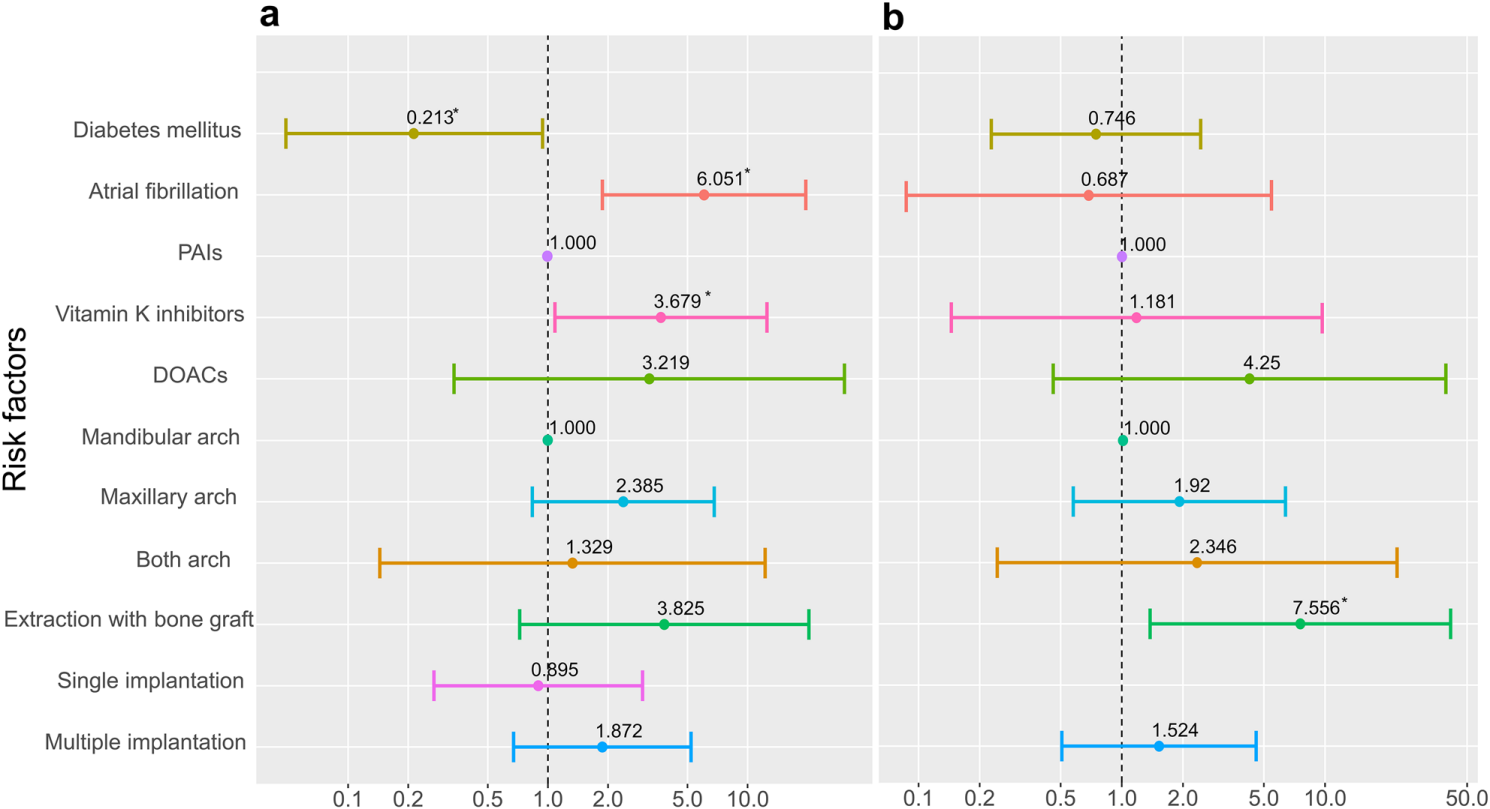
Subgroup comparison of odds ratio for the associated risk factors: (**a**) the maintenance group and (**b**) the discontinuation group. The 95% confidence interval and odds ratio for each variable is presented. Statistically significance observed in the univariate analyses is indicated by asterisk *(*p* < 0.05).

The findings of intergroup comparisons of risk factors related to post-operative bleeding within the two subgroups are reported in Supplementary Table S1. Univariate analyses were performed to identify the significant bleeding risk factors (Table 4). In the maintenance group, statistical significance was found for diabetes mellitus (OR = 0.213, *p* = 0.041), atrial fibrillation (OR = 6.051, *p* = 0.003) and vitamin K inhibitors (OR = 3.679, *p* = 0.037). Performing extraction with bone grafting (OR = 7.556, *p* = 0.020) had a significant impact on the post-operative bleeding rate in the discontinuation group. The data for the main risk factors are summarized graphically in Fig. 2.

**Table 4:** Univariate analyses of possible risk factors for post-operative bleeding events in subgroups: the maintenance group and the discontinuation group.

	Maintenance group (n = 245)				Discontinuation group (n = 292)			
	OR	95% Confidence Interval		*p*-value	OR	95% Confidence Interval		*p*-value
		Lower	Upper			Lower	Upper	
Age	0.987	0.942	1.035	0.593	0.967	0.920	1.016	0.180
Sex								
Male	1 (Ref)				1 (Ref)			
Female	1.234	0.504	3.025	0.645	2.268	0.741	6.942	0.151
Smoking history								
None	1 (Ref)				1 (Ref)			
Former smoker	3.333	0.644	17.245	0.151	2.901	0.332	25.358	0.336
Current smoker	1.667	0.194	14.306	0.641	NA	NA	NA	0.992
Presence of systemic diseases								
Hypertension	0.521	0.212	1.283	0.156	0.583	0.189	1.799	0.348
Diabetes mellitus	0.213	0.048	0.940	0.041*	0.746	0.228	2.442	0.629
Cerebrovascular diseases	NA	NA	NA	0.990	0.517	0.066	4.073	0.531
Osteoporosis	1.362	0.431	4.302	0.599	2.451	0.732	8.203	0.146
Atrial fibrillation	6.051	1.873	19.553	0.003*	0.687	0.087	5.449	0.722
Artificial heart valves	NA	NA	NA	1.000	1.738	0.525	5.761	0.366
Deep vein thrombosis	0.572	0.073	4.513	0.596	2.407	0.500	11.586	0.273
Myocardial infarction	2.842	0.563	14.346	0.206	0.853	0.107	6.812	0.881
Pulmonary embolisms	5.550	0.482	63.910	0.169	NA	NA	NA	0.992
Anticoagular agents								
PAIs	1 (Ref)				1 (Ref)			
Vitamin K inhibitors	3.679	1.084	12.486	0.037*	1.181	0.145	9.594	0.877
DOACs	3.219	0.339	30.525	0.308	4.250	0.460	39.276	0.202
Dental arch								
Mandibular arch	1 (Ref)				1 (Ref)			
Maxillary arch	2.385	0.836	6.801	0.104	1.920	0.577	6.388	0.288
Both	1.329	0.144	12.224	0.802	2.346	0.244	22.589	0.460
Type of teeth								
Anterior	1 (Ref)				1 (Ref)			
Posterior	1.037	0.289	3.717	0.956	1.381	0.300	6.356	0.679
Type of surgery								
Flap operation	1 (Ref)				1 (Ref)			
Extraction	0.441	0.118	1.645	0.223	1.571	0.304	8.112	0.589
Implant surgery	0.889	0.311	2.546	0.827	0.880	0.172	4.515	0.878
Periodontal flap operation								
Type of periodontal flap operation								
One sextant	1.261	0.396	4.018	0.695	1.219	0.261	5.696	0.801
Multiple sextants	1.944	0.398	9.507	0.412	NA	NA	NA	0.990
Teeth extraction								
Type of teeth extraction								
One tooth	0.360	0.080	1.614	0.182	1.865	0.541	6.437	0.324
Multiple teeth	0.706	0.154	3.244	0.654	1.516	0.308	7.462	0.609
Number of teeth involved (extraction)	0.818	0.496	1.348	0.430	1.078	0.722	1.609	0.713
Extraction with bone graft	3.825	0.722	20.267	0.115	7.556	1.378	41.422	0.020*
Hemostatic filler	0.261	0.034	2.008	0.197	0.992	0.214	4.597	0.991
Implant surgery								
Type of implant surgery								
Single implantation	0.895	0.269	2.977	0.856	NA	NA	NA	0.988
Multiple implantation	1.872	0.673	5.209	0.230	1.524	0.507	4.581	0.453
Combined sinus augmentation								
Crestal approach	1.340	0.287	6.265	0.710	1.976	0.413	9.461	0.394
Lateral approach	5.694	0.492	65.879	0.164	4.545	0.489	42.286	0.183
Combined ridge augmentation	1.917	0.770	4.771	0.162	1.422	0.479	4.216	0.526
Hemostatic measures								
Bite swab	1 (Ref)				1 (Ref)			
Hemostatic filler and suturing	0.306	0.026	3.571	0.345	0.800	0.068	9.452	0.859
Bite swab and suturing	1.193	0.259	5.492	0.821	0.793	0.096	6.533	0.829

Odds ratios were denoted as ‘NA’ under circumstances incapable of deriving values: (1) no patients were included in certain risk factors; (2) the bleeding ratios did not differ depending on certain risk factors. For systemic conditions, a reference value of 1 was set for cases having no systemic diseases. Regarding the treatment-related parameters of each dentoalveolar surgery, cases where the surgery or additional procedure was not performed served as reference.

*Statistically significant (*p* < 0.05).

### Multiple logistic regression analysis

The parameters that were significant in statistical analyses were included as explanatory variables in the multivariate logistic regression analysis: diabetes mellitus, atrial fibrillation, the type of anticoagulant agents, the dental arch involved, extraction with bone graft and the extent of implant surgery (single or multiple). Stepwise selection method was used for the analysis, and the variables that did not fit the model significantly were excluded.

The results showed that all explanatory variables except systemic conditions (diabetes mellitus and atrial fibrillation history) have been shown to exhibit significant effects on the post-operative bleeding (Table 5). The vitamin K inhibitors (OR = 4.154, *p* = 0.014), DOACs (OR = 6.422, *p* = 0.040), involvement of the maxillary arch (OR = 2.414, *p* = 0.036), extraction with bone grafting (OR = 6.766, *p* = 0.005) and multiple implantations (OR = 2.633, *p* = 0.024) were significant risk factors for post-operative bleeding events.

**Table 5:** Multivariate logistic regression analysis of post-operative bleeding events.

Variables	OR	95% Confidence Interval		*p*-value
		Lower	Upper	
Diabetes mellitus	0.433	0.169	1.112	0.082
Atrial fibrillation	2.408	0.881	6.578	0.087
Anticoagular agents				
PAIs	1 (Ref)			
Vitamin K inhibitors	4.154	1.341	12.870	0.014*
DOACs	6.422	1.091	37.791	0.040*
Dental arch				
Mandibular arch	1 (Ref)			
Maxillary arch	2.414	1.058	5.510	0.036*
Both	1.024	0.173	6.049	0.979
Extraction with bone graft	6.766	1.794	25.521	0.005*
Type of implant surgery				
Single implantation	0.417	0.130	1.334	0.140
Multiple implantation	2.633	1.134	6.112	0.024*

For systemic conditions, a reference value of 1 was set for cases having no systemic diseases. Regarding the treatment-related parameters of each dentoalveolar surgery, cases where the surgery or additional procedure was not performed served as reference.

*Statistically significant (*p* < 0.05).

## Discussion

Post-operative bleeding is one of the main patient-reported outcomes associated with invasive dental treatments. The increasing number of patients receiving anticoagulant therapies raises concerns about medication withdrawal prior to dentoalveolar surgeries. Numerous previous studies have recommended continuing medication in patients receiving anticoagulant therapies, as the thromboembolic risk associated with medication withdrawal outweighs the benefits of preventing post-operative bleeding. Furthermore, for commonly used medications such as PAIs and vitamin K inhibitors, it has been shown that intra- and post-operative bleeding events can be sufficiently controlled by local haemostatic management.

However, several challenges hinder investigations of post-operative bleeding in dentoalveolar surgeries. Most studies have assessed the bleeding risk focusing on various local haemostatic measures performed after dental procedures, thereby making it difficult to solely evaluate the post-operative bleeding risk of dentoalveolar surgeries. Moreover, the definitions of bleeding events have varied among studies, and most bleeding management decisions are made at the discretion of clinicians. A recent systematic review has documented the difficulty of obtaining meaningful findings on this issue^10^. Also, risk classifications for peri-operative bleeding have been attempted based on the invasiveness and extent of dentoalveolar surgeries^11^. In contrast, there is a lack of research addressing the association between post-operative bleeding and risk factors related to invasive dental treatments.

These factors have led to the current situation where no specific guidelines exist for dental clinicians to refer to when they encounter patients with a high bleeding risk, and the available recommendations primarily advise maintaining anticoagulants whenever possible. Case-specific considerations and the establishment of appropriate guidelines are needed. The present study is the first to examine the association of systemic and clinical factors in patients receiving anticoagulant therapies with post-operative bleeding, and to identify patient- and treatment-related factors for which medication continuation may be unfavourable.

The incidence of post-operative bleeding has been reported to range between 0 and 26%^10^. The current study found a relatively low rate of 6.52% bleeding events. It is assumed that this lower rate can be attributed to the bleeding events being recorded after patients report bleeding complications during post-operative visits, rather than being recorded based on specific criteria or by designated staff members.

Numerous studies support the recommendation that discontinuing antiplatelet therapy is unnecessary in patients taking anticoagulants^11^. However, the evidence remains insufficient for vitamin K inhibitors and DOACs. The RR of post-operative bleeding due to the maintenance of vitamin K inhibitors was reported to be only around 6%^3^. A recent prospective research study found that the incidence of post-operative bleeding was significantly higher when vitamin K inhibitors were maintained (11.3%) than in the PAI group (0.8%) and in the control group without anticoagulant therapies (0.7%)^7^. The current study found that the bleeding rate varied significantly with the medication being taken, with higher bleeding rates observed in the vitamin K inhibitor group (13.5%) and the DOAC group (22.2%), although the differences were not statistically significant in post-hoc analysis (Table 2). The subgroup analysis confirmed that vitamin K inhibitors were a significant risk factor in the maintenance group (Table 4). This underscores the importance of exercising caution when dental procedures are performed in patients taking vitamin K inhibitors.

The distribution of patient- and treatment-related factors generally did not differ significantly between the maintenance and discontinuation groups, with the exception of the systemic conditions of hypertension and the presence of an atrial heart valve (Supplementary Table 1). This suggests that evaluating the effect of anticoagulant continuation by comparing these two subgroups is feasible; however, the results should be interpreted with caution, particularly for patients with hypertension or the presence of an artificial heart valve.

We found that bleeding rate was 8.57% in the maintenance group and 4.79% in the discontinuation group. Although statistical significance was not observed regarding medication withdrawal, the univariate regression analyses (see Table 4) identified several statistically significant risk factors only within the maintenance group (atrial fibrillation and vitamin K inhibitor). This implies the need for greater consideration of systemic factors when undertaking invasive treatments in patients taking anticoagulants.

The multivariate analysis (see Table 5) identified that several treatment factors increased the bleeding risk: involvement of the maxillary arch (*p* = 0.036), extraction with bone grafting (*p* = 0.005) and multiple implantations (*p* = 0.024). Recent studies have suggested that extracting multiple teeth can significantly increase the bleeding risk^12–14^. While the present study did not produce evidence of a significant relationship between the extent of flap elevation or tooth extraction and the bleeding risk, it did find that the bleeding risk was higher for multiple implantations (Table 2). The only previous study of implant therapies found no significant effect of the number of implants on post-operative bleeding^6^. Since there have been only a small number of studies on the extent of implant surgery and post-operative bleeding, further research with larger cohorts is needed to evaluate the effect of multiple implantations.

From the perspective of augmentation procedures, extraction with bone grafting was a critical factor related to a significantly increased post-operative bleeding risk in this study, as observed in various statistical analyses including subgroup analyses (Tables 2, 3, 4 and 5). Extraction with bone grafting, also known as alveolar ridge preservation, is generally considered a reliable and stable technique for bone regeneration. However, few studies have addressed post-operative complications, including post-operative bleeding. A recent systematic review found that post-operative bleeding was one of main post-operative complications, although that review only included a small number of relevant studies^15^. Wound stabilization, which is considered to be associated with the presence of bone grafting materials and the surgical site, may have contributed to post-operative bleeding by interfering with early clot formation. Further studies addressing the relationships between bone augmentation procedures and post-operative bleeding events are needed.

Regarding implant therapies, a previous study found that implant surgery-related factors (i.e. implant exposure, ridge augmentation and sinus augmentation) were not significantly related to the post-operative bleeding rate^6^, which was consistent with the findings of the present study. A recent literature review also did not find any significant association between implant surgery and post-operative bleeding, though it was noted that only a small number of studies evaluated the bleeding risk related to augmentation procedures such as sinus augmentation and guided bone graft^16^.

The present study found no significant effects of intraoperative haemostatic measures. Most studies have suggested that patients receiving anticoagulant therapies , when provided with appropriate local haemostatic management immediately after surgery, experience minimal post-operative bleeding events and no fatal events due to bleeding^2,17,18^. Also in our study, all patients who experienced post-operative bleeding were successfully managed with appropriate local haemostatic measures, and no morbidity was observed. There is a lack of prior research into the efficacy of local haemostatic measures, probably because most haemostatic interventions are performed based on the judgement of clinicians regarding the intraoperative status.

It is known that medication withdrawal increases the risk of thromboembolic events by more than threefold^19^. Hence, conducting a randomized study to determine the maintenance or withdrawal of medication in patients receiving anticoagulant therapies is considered unethical. However, a recent meta-analysis investigating post-operative bleeding in patients receiving anticoagulant therapies found a significantly higher relative risk (RR = 2.794) than those not receiving anticoagulant therapies^20^. This indicates that patients receiving anticoagulant therapies have a clearly increased bleeding risk, and caution is required when treating patients with factors that may further increase the bleeding risk. For patients with a history of atrial fibrillation or who are taking vitamin K inhibitors, it is advisable to reconsider the maintenance of anticoagulants or to exercise caution when implementing local haemostatic measures (Table 4).

This study had several limitations. First, its retrospective design meant that there is a possibility of bias in patient inclusion and the composition of the subgroups. Second, the sample sizes differed among patients using the different types of anticoagulant. The numbers of patients taking vitamin K inhibitors (*n* = 37) and DOACs (*n* = 11) in this study were markedly smaller than those taking PAIs (*n* = 489). The low numbers of vitamin K inhibitors and DOACs may have led to the overestimation of certain findings where statistical significance was achieved. Since the mechanisms of action vary among anticoagulants, the comprehensive interpretation of results may obscure specific findings detectable for each medication. Moreover, detailed information of the medications such as the dosage and half-life had not been included in this study. This should as well be addressed as a shortcoming of the study since these properties have been shown to influence the post-operative bleeding risk^21^. Regarding DOACs, a recent review has recommended performing dental surgeries 12–24 h after last intake, considering the half-life of medications^21^. For the vitamin K inhibitors, bridging with low molecular weight heparins is suggested to minimize the thromboembolic risk after discontinuation of medications, though no definitive guidelines have been provided^4^. These specific approaches for certain types of anticoagulants should be taken into account. Third, the occurrence of post-operative bleeding events was detected primarily based on patient reports, so there might have been post-operative bleeding events that were not included in the analyses. Further prospective studies that include the documentation of INR values should be carried out.

Within the limitations of the study, we conclude that patients with maintenance of anticoagulants showed comparable post-operative bleeding rate to patients with discontinuation of anticoagulants (*p* = 0.081) (Table 3). Vitamin K inhibitors and atrial fibrillation may increase the post-operative bleeding rate in patients taking anticoagulants. Since several treatment factors (extraction with bone grafting, multiple implantations and involvement of the maxillary arch) were associated with higher risks of post-operative bleeding, it is recommended to consider continuing anticoagulants in minimally invasive dentoalveolar surgeries along with appropriate medical consultation.

### Supplementary Information

Supplementary Table S1.

## Data Availability

The data that support the findings of this study are available on request from the corresponding author. The data are not publicly available due to privacy or ethical restrictions.
