# Supplementary material for: Risk of post-operative bleeding after dentoalveolar surgery in patients taking anticoagulants: a cohort study using the common data model
Source: Sci Rep. 2024 Apr 2;14:7787. doi: 10.1038/s41598-024-57881-7 (PMC10987490; doi:10.1038/s41598-024-57881-7)
Supplement: Supplementary file 1 — Supplementary Table S1. [file 41598_2024_57881_MOESM1_ESM.pdf]

Risk of post-operative bleeding after dentoalveolar surgery in patients taking anticoagulants: a cohort study using the common data model

Joo-Yeon Lee[1,*], Seung-Hyun Park[1,*], Da-Mi Kim[1], Kyung-A Ko[1], Jin-Young Park[1], Jung-Seok Lee[1], Ui-Won Jung[1], Jae-Kook Cha[1,#]

[1]Department of Periodontology, Research Institute for Periodontal Regeneration, Yonsei University College of Dentistry, Seoul, Republic of Korea

[*]These authors contributed equally to this work.

[#]Corresponding author: Jae-Kook Cha

Department of Periodontology, Research Institute for Periodontal Regeneration, Yonsei University College of Dentistry, 50 Yonsei-ro, Seodaemun-gu, Seoul 03722, Republic of Korea

Tel.: +82-2-22283191

Fax: +82-2-3920398

E-mail: chajaekook@gmail.com

Supplementary Table 1. Overall demographic characteristics of patients taking anticoagulants.

	Maintenance group (*n*=245)	Discontinuation group (*n*=292)	Total	*p*-value
Age (mean ± SD)	70.70±9.28	71.26±9.89	71.0±9.63	.504
Sex, *n* (%)				.647
male	140 (57.14)	160 (54.79)	300 (55.87)	
female	105 (42.86)	132 (45.21)	237 (44.13)	
Smoking history, *n* (%)				.680
none	228 (93.06)	277 (94.86)	505 (94.04)	
former smoker	9 (3.67)	8 (2.74)	17 (3.17)	
current smoker	8 (3.26)	7 (2.40)	15 (2.79)	
Systemic diseases, *n* (%)				
Hypertension	163 (66.53)	219 (75)	382 (71.14)	**.039***
Diabetes mellitus	76 (31.02)	101 (34.59)	177 (32.96)	.433
Cerebrovascular diseases	27 (11.02)	37 (12.67)	64 (11.92)	.650
Osteoporosis	37 (15.10)	43 (14.73)	80 (14.90)	.999
Atrial fibrillation	16 (6.53)	29 (9.93)	45 (8.38)	.208
Artificial heart valves	70 (28.57)	56 (19.18)	126 (23.46)	**.014***
Deep vein thrombosis	19 (7.76)	20 (6.85)	39 (7.26)	.814
Myocardial infarction	10 (4.08)	24 (8.22)	34 (6.33)	.075
Pulmonary embolisms	3 (1.22)	8 (2.74)	11 (20.05)	.353
Anticoagular agents, *n* (%)				.929
PAIs	222 (90.61)	267 (91.44)	489 (91.06)	
Vitamin K inhibitors	18 (7.35)	19 (6.51)	37 (6.89)	
DOACs	5 (2.04)	6 (2.05)	11 (2.05)	
Dental arch, *n* (%)				.658
mandibular arch	98 (40)	126 (43.15)	224 (41.71)	
maxillary arch	132 (53.88)	152 (52.05)	284 (52.89)	
both	15 (6.12)	14 (4.79)	29 (5.40)	
Type of teeth, *n* (%)				.290
anterior	36 (14.69)	54 (18.49)	90 (16.76)	
posterior	209 (85.31)	238 (81.51)	447 (83.24)	
Type of surgery, *n* (%)				.090
flap operation	55 (22.45)	46 (15.75)	101 (18.81)	

extraction	78 (31.84)	90 (30.82)	168 (31.28)	
implant surgery	112 (45.71)	156 (53.42)	268 (49.91)	
Periodontal flap operation (*n* = 101)				.141
one sextant	41 (16.73)	34 (11.64)	75 (13.97)	
multiple sextants	14 (5.71)	12 (4.11)	46 (8.57)	
Teeth extraction (*n* = 168)				
Type of teeth extraction				.885
one tooth	51 (20.82)	56 (19.18)	107 (19.93)	
multiple teeth	27 (11.02)	34 (11.64)	61 (11.36)	
Number of teeth involved (extraction) (mean ± SD)	1.88± 1.72	1.73± 1.44		.835
Extraction with bone graft, *n* (%)				.919
Yes	8 (3.27)	8 (2.74)	16 (2.98)	
No	70 (28.57)	82 (28.08)	152 (28.31)	
Use of hemostatic filler, *n* (%)				.911
Yes	37 (15.10)	42 (14.38)	79 (14.71)	
No	41 (16.73)	48 (16.44)	89 (16.57)	
Implant surgery (*n* = 268)				
Type of implant surgery, *n* (%)				.181
single implantation	59 (24.08)	87 (29.79)	146 (27.19)	
multiple implantation	53 (21.63)	69 (23.63)	122 (22.72)	
Combined sinus augmentation, *n* (%)				.720
none	90 (36.73)	125 (42.81)	215 (40.04)	
crestal approach	19 (7.76)	25 (8.56)	44 (8.19)	
lateral approach	3 (1.22)	6 (2.05)	9 (1.68)	
Combined ridge augmentation, *n* (%)				.202
Yes	72 (29.39)	102 (34.93)	174 (32.40)	
No	40 (16.33)	54 (18.49)	94 (17.50)	
Hemostatic measures, *n* (%)				.204
bite swab	24 (9.80)	17 (5.82)	41 (7.64)	
hemostatic filler and suturing	37 (15.10)	42 (14.38)	79 (14.71)	
bite swab and suturing	184 (75.10)	233 (79.79)	417 (77.65)	

*(bold): statistically significant in chi-square test or fisher test ($p < 0.05$).
